# Challenges and Successes of Pregnancy Assistance Fund Programs Supporting Young Fathers

**DOI:** 10.1007/s10995-020-02923-5

**Published:** 2020-04-24

**Authors:** Katherine Niland, Rebekah Selekman

**Affiliations:** Mathematica Policy Research, Cambridge, USA

**Keywords:** Young father, Fatherhood, Parenting teen, Expectant and parenting youth

## Abstract

**Introduction:**

Since 2010, the Office of Population Affairs (formerly the Office of Adolescent Health) has offered funding to support expectant and parenting youth through the Pregnancy Assistance Fund (PAF). PAF grantees typically focus on serving young mothers, so programs serving young fathers are more limited.

**Methods:**

Based on a purposive sample of nine past and current PAF grantees that serve young fathers, this study describes the overall program designs, provides a detailed view of the challenges to serving young fathers, and identifies key strategies for successful father engagement.

**Results:**

Across grantees, program components were largely the same for both young fathers and mothers. Recruitment and engagement were the most commonly cited challenges to young fathers’ participation in the PAF-funded programs.

**Discussion:**

Successful strategies for serving young fathers included recruiting from places that fathers naturally frequent, creating program space welcoming to fathers, and hiring staff who understand the experiences of young fathers.

## Significance

Several studies have demonstrated that father involvement with young mothers and their children benefits all parties. Research on how programs engage with young fathers to improve family involvement is limited. This study describes current and past PAF grantee approaches to recruiting and serving young fathers. The findings of this exploratory study aim to highlight these programs’ experiences serving young fathers and contribute to the currently limited research available on this topic.

## Introduction

Several studies demonstrate the importance of father involvement for young mothers and their children, though research also documents the challenges that young fathers face to being involved with their families (Amato and Gilbreth 1999; Tamis-LeMonda et al. 2004; McWayne et al. 2013). The family benefits of father involvement include enhanced social and academic development in children and improvements in mothers’ receipt of prenatal care (Bronte-Tinkew et al. 2008; National Responsible Fatherhood Clearinghouse 2007; Alio et al. 2011; Martin et al. 2007; Howard et al. 2006). However, challenges related to poverty, relationships, and criminal justice involvement make it difficult for low-income fathers to be as engaged as they may want to be in the lives of their children (Bryan 2013; Edin and Nelson 2013; Mincy et al. 2016; Carlson et al. 2017).

Since 2010, the Office of Adolescent Health has offered funding to support and improve outcomes for expectant and parenting youth through the Pregnancy Assistance Fund (PAF). The first cohort of PAF grantees, whose awards began in 2010, focused efforts on providing services including childcare, education, and flexible academic scheduling to pregnant and parenting mothers in high school or institutes of higher education. Since 2013, PAF grantees have been expected, but not required, to offer services for young fathers. In 2019, the federally funded PAF program provided support to 23 grantees, including 22 states and one tribal organization (U.S. Department of Health and Human Services 2018). According to the most recent PAF Performance Measures Report, young fathers represented about 14% of all youth served with PAF funds between 2017 and 2018 (U.S. Department of Health and Human Services 2019).

Though much research exists about the importance of fathers in the lives of children and families, we find less documentation of how programs that serve young parents engage with young fathers to improve their family well-being. This study aims to address this gap by describing the program designs of a sample of PAF programs serving young fathers, providing a detailed view of the challenges to serving this population, and identifying key strategies for successfully serving young fathers. The findings of this study highlight current and past PAF programs’ experiences serving young fathers and contribute to the limited research available on this topic. The successful strategies highlighted in this study may be helpful to researchers or practitioners who wish to serve this important population but face similar challenges developing or implementing programs.

## Methods

The study team conducted phone interviews with a purposive sample of nine past and current PAF grantees. We recruited grantees that (1) either offered specific services for young fathers or otherwise targeted young fathers for services and (2) demonstrated an increasing number of young fathers served each year. We identified 10 grantees that met the study eligibility criteria; 9 agreed to participate in the semi-structured interviews. Interview questions covered several topics, including identifying, recruiting, and serving young fathers; overseeing programs for young fathers; and assessing the perceived effectiveness of PAF-funded programs. Grantee program representatives recruited to be respondents included program or service directors and coordinators whose responsibilities involved overseeing the PAF grant and overall program operations. Prior to the beginning of the interview, all respondents from participating grantee sites were informed that the information they shared would be part of a special article series for the *Maternal and Child Health Journal* before they consented. The sample included past grantees (New Hampshire [funded 2015–2018] and New Jersey [funded 2013–2018]) and current grantees (Connecticut, Virginia, New Mexico, and Montana [funded 2010–present] and Michigan, South Carolina, and New York [funded 2013–present]). Participating grantees included one that served young fathers and mothers separately and eight that served young mothers and fathers together.

## Results

The study team imported notes from each interview into qualitative research software NVivo 12. The primary author developed a coding structure consisting of 37 categories and subcategories. Interview notes were coded by the primary author and one trained coder; the trained coder also assisted as a note taker for the interviews. Our analysis yielded three major findings: (1) PAF grantees’ overall initial program design, including the use of case management services and group-based education using evidence-based curricula, was largely the same for both mothers and fathers; (2) programs identified unique challenges to recruiting and serving young fathers; (3) during implementation, programs applied a few key strategies to successfully serve young fathers. Our analysis revealed other challenges for programs serving young parents; however, these were not unique to programs serving fathers. Such challenges included the need for a network of partners to support referrals and services, the need for adequate funding to ensure program sustainability, and the need for qualified staff to increase the capacity to serve the full eligible population. Findings specific to the experiences of programs serving young fathers are discussed in detail in the sections below.

### Similar Program Design

Grantees designed their PAF programs to meet the funding objectives and improve outcomes for participants. Grantees’ program objectives were primarily to support fathers’ financial independence and more involvement in their child’s lives. Grantee outcomes for participants were the same for young mothers and young fathers. Table 1 displays the entire menu of available services offered by each PAF program. The table does not distinguish services for fathers from services for mothers because, for the most part, grantees designed their service array to be available to all participants. In this section, we discuss program components as designed, prior to implementation. In the section describing strategies for recruitment and engagement, we discuss changes made to the service array during implementation.

**Table 1:** Components of PAF programs serving young fathers, by state

	Connecticut	Michigan	Montana	New Hampshire	New Jersey	New Mexico	New York	South Carolina	Virginia	Total PAF
Available services										
Parenting services	✓	✓	✓	✓	✓	✓	✓	✓	✓	9
Employment services	✓	✓	✓	✓	✓	✓	✓	✓	✓	9
Child care	✓	✓	✓	✓	✓	✓			✓	6
Flexible or alternative academic scheduling	✓		✓	✓	✓					4
Transportation	✓	✓					✓		✓	4
Health care	✓		✓					✓		3
Mental health counseling				✓	✓					2
Family counseling relationship building				✓						1
Transition for postsecondary education	✓									1
Mode of service delivery										
Referral services	✓	✓	✓	✓	✓	✓	✓	✓	✓	9
Case management	✓	✓	✓	✓	✓	✓	✓	✓	✓	9
Group-delivered curriculum/workshops	✓	✓	✓	✓	✓	✓	✓	✓	✓	9
Home visiting		✓	✓		✓	✓			✓	5
Support groups		✓					✓		✓	3
Total number of components	10	9	9	8	9	7	7	6	9	

We populated this table based on a review of grantee materials and interview responses. The findings presented in this report update prior descriptions of PAF program components (for example, Person et al. 2018). Any inconsistencies across reports may be due to changes in programming over time and across grant periods, reflecting, for example, changes in emphasis in the Funding Opportunity Announcements

All respondents described that their programs provided services to both fathers and mothers. All programs used case managers to assign participants to services offered by the program. Services were typically delivered through group-based classes or workshops. Some programs offered the group-based classes to fathers and mothers separately, and other programs tailored their classes for fathers and mothers to attend together. Five of the nine programs also provided services via home visiting. One-on-one counseling was only used by two of the nine programs. If there were service needs that the grantee’s program could not meet, case managers would make referrals to other service providers. Six of the nine programs also made childcare available to program participants. Regardless of the particular components selected, all respondents reported designing their programs based on evidence from research or best practices.

As designed, programs offered incentives for participation or program completion that were the same for mothers and fathers. Types of incentives offered varied across programs but included food; transportation; gift cards; items for children including diapers, wipes, and car seats; and incentives to promote parent–child relationships, such as tickets to the zoo. We discuss how programs adapted available incentives for fathers in the next section on strategies for recruitment and engagement.

### Distinct Challenges to Serving Young Fathers

Although the design of PAF-funded programs was largely similar for young fathers and mothers, respondents reported several distinct challenges to serving fathers, especially with respect to recruitment and engagement. A central challenge to recruiting fathers is the difficulty of identifying them. About expectant young mothers, one respondent said, “They’re physically visible,” whereas expectant fathers are not. Moreover, connecting fathers to PAF-funded services can be challenging because young fathers are often disconnected from broader systems of care. There can be multiple reasons for this disconnection. One reason is young fathers are often not the custodial parent, restricting their eligibility for many services. Respondents offered that fathers might also feel stigma for participation in services—that their participation would label them as “absent” fathers. This stigma causes fathers to further disconnect from systems of care. As one PAF grant coordinator described, “Mothers, I think, are used to sharing and being connected socially with other people and wanting to find those spaces [of] warmth, whereas fathers have been excluded from that process of parenting, excluded from programs, excluded from the system.”

Grantees suggested that another unique challenge to engaging fathers in services is the societal pressure young fathers feel to be employed and earn money to provide for their family or make child support payments. This can create competing priorities for young fathers, forcing them to choose between their financial responsibilities to their children and investment in their own education or other long-term goals. It can also make it difficult for fathers to find time to participate in programs.

### Key Strategies for Father Recruitment and Engagement

Although most programs had a singular program design prior to implementation, regardless of whether the participants were mothers or fathers, most grantees adapted their programs during implementation to better recruit and engage young fathers. Key strategies reported by grantees included hiring male staff, employing creative recruitment approaches, developing a more welcoming environment for fathers, and offering services that explicitly cater to fathers.

- More than half of the grantees (five of the nine) reported hiring male fatherhood specialists, and they reported that males were more effective than females at recruiting and retaining fathers in the program, especially if the specialists were fathers themselves or were closer in age to the program participants. One grantee remarked that one program site, which employed a male social worker, was able to recruit more young fathers than in areas without a male staff member. The respondent described that as time went on, they began to realize that “who was delivering messages mattered; men want to talk to men.” Employing staff that understand the experiences of young fathers is key to “meeting fathers where they’re at” and successfully recruiting them.
- Creative recruitment and outreach strategies included reaching out to juvenile correctional facilities and court systems, as well as targeting young men in places they frequent, such as gyms, barbershops, and community centers.
- Grantees also mentioned that creating a comfortable and inviting space for fathers was key to successfully engaging fathers. For instance, grantees developed printed program materials geared specifically toward fathers and featured pictures of fathers on program brochures or posters on the wall. Other strategies for engaging fathers included having game nights with food, pool tables, televisions, and video games.
- Grantees also adapted their service components in order to develop a service array that was engaging and appropriate for fathers. Common adaptations included offering events where fathers could deepen their relationship with their child; offering job placement services to help them achieve financial independence and support their child; incentives aimed at helping fathers obtain employment, including haircuts for interviews or gift cards to purchase work clothing; creating fathers-only groups; and using father-specific curricula, such as 24/7 Dads and Nurturing Fathers.

## Discussion

Despite the particular challenges to serving young fathers, all grantees believed the young men benefited from their programs. Although most grantees did not analyze program data specifically to examine father engagement, grantees described that their programs increased fathers’ access to resources to support their education, financial stability, and involvement in their child’s lives.

Given the exploratory nature of this study, we prioritized recruiting respondents who understood the broader context of the PAF grant and overall program operations, such as program directors. The study is limited because it lacks the perspectives of service delivery staff and fathers themselves. Nevertheless, this study fills an important gap in research by describing the characteristics and experiences of PAF grantees. Future research could build on this study by examining fathers’ outcomes after participating in programs, by gathering data from more and varied programs serving young fathers, and by conducting in-depth interviews with program participants about their own experiences and perceptions of effective engagement strategies. Another recent study published in this supplement highlights the perspectives of both fathers and staff on program engagement (McGirr et al. in this issue).

Based on the findings of this study, future grantees might consider designing or adapting their programs to address the distinct challenges related to serving young fathers. Attention to the issues raised here may help bolster recruitment and engagement of this important population. This study suggests some key strategies that other PAF grantees and non-PAF programs could use to serve this population. Building these strategies into the program design, and enhancing the way in which programs serve fathers, can have important benefits for father, child, and family well-being (U.S. Department of Health and Human Services 2004; The Fatherhood Project 2014).
